# Laparoscopic Versus Open Appendectomy for Patients With Perforated Appendicitis

**DOI:** 10.7759/cureus.26265

**Published:** 2022-06-23

**Authors:** Sayed Farhad Rasuli, Jasmeen Naz, Najim Azizi, Nabeel Hussain, Pir Naveed Ahmed Ahsan Qureshi, Kiran Maee Swarnakari, Wahidullah Dost, Shumaila Zafar, Laila Tul Qadar, Abdul Subhan Talpur

**Affiliations:** 1 General Surgery, Liaquatian Academic and Research Society, Hyderabad, PAK; 2 Plastic and Reconstructive Surgery, Liaquat University of Medical & Health Sciences, Hyderabad, PAK; 3 Internal Medicine, The University of Lahore, Lahore, PAK; 4 Gynecology and Obstetrics, Mayo Hospital, Lahore, PAK; 5 Internal Medicine, Dow University of Health Sciences, Civil Hospital Karachi, Karachi, PAK; 6 Medicine, Liaquat University of Medical & Health Sciences, Jamshoro, PAK

**Keywords:** laparascopic surgery, perforated appendicitis, laparoscopic, appendectomy, acute pancreatitis

## Abstract

Introduction

Acute appendicitis can lead to perforation which can be lethal. The present study assessed the outcomes of laparoscopic appendectomy versus open appendectomy in patients with perforated appendicitis.

Methodology

A comparative study was conducted at the Department of Surgery, Liaquat University of Medical & Health Sciences (LUMHS), between March 2, 2019 and July 7, 2020. The inclusion criteria consisted of a diagnosis of perforated appendicitis. Exclusion criteria consisted of intellectual disability hindering the procurement of informed consent, pediatric patients < 15 years of age, patients with an appendicular mass or abscess unsuited for laparoscopic appendectomy, severe sepsis or septic shock on presentation, and pregnant women. Patients were allocated to either open appendectomy (Group A) or laparoscopic appendectomy (Group B). The data gathering proforma recorded demographics, surgical findings, operating room (OR) time, length of inpatient care, requirement of analgesic, and any adverse events following surgery. All of the surgeries were conducted by an experienced surgical consultant with an experience of at least five years.

Results

A total of 85 patients were included in the laparoscopic appendectomy group, while 101 cases were included in the open appendectomy group. The use of analgesics thrice a day to manage the postoperative pain was significantly associated with the open appendectomy (p < 0.0001). Moreover, the postoperative length of hospitalization was substantially greater in patients who underwent open appendectomy than those who underwent laparoscopic procedure (p < 0.0001). Wound-related complications were considerably lower in patients who had laparoscopic appendectomy as compared to those who had open appendectomy (23.53% versus 40.5%; p = 0.013).

Conclusion

The length of stay was significantly lower in patients who underwent laparoscopic appendectomy. Moreover, laparoscopic appendectomy was also associated with a lower rate of wound infection postoperatively, thus giving the former an edge over the latter. Despite the finding that the postoperative pain was not considerably different between the two groups, patients who underwent open appendectomy group required significantly more painkillers to manage the postoperative pain.

## Introduction

A dreaded consequence of acute inflammation of the appendix is perforation [1]. Perforation is even more likely if the inflammation is associated with impaction of fecal matter within the appendix (fecalith) and development of a peri-appendiceal abscess [1,2]. Perforation approximately occurs in up to 30% of patients suffering from appendicitis [3]. 76% of patients present to the hospital with a triad of pain, vomiting, and fever [4].

Appendicitis is classically managed with open appendectomy though newer guidelines also support minimally invasive laparoscopic intervention in uncomplicated cases [4-6]. The advantages of laparoscopy over open surgery have been clearly established [6]. Open appendectomy is associated with increased surgical site infections (SSIs), incidence of incisional hernias, and wound dehiscence. Perforated appendicitis is classically managed with open appendectomy and there is lacking evidence regarding the use of laparoscopic appendectomy. Perforated appendicitis itself is associated with negative outcomes such as higher rates of morbidity and lengthier inpatient stay [6,7].

There are very few studies that compare the laparoscopic appendectomy with open appendectomy in patients and deduce which one is an appropriate and useful surgical intervention for perforated appendicitis. Local studies are limited and many of these studies have not been conducted on a sample size that may produce significant evidence and allow actionable change and so it remains undetermined whether laparoscopic appendectomy has advantages over open appendectomy for perforated appendicitis. 

One significant benefit of laparoscopic appendectomy over open appendectomy is that the former allows for direct visualization of the peritoneum as it is washed to prevent peritonitis [8]. Laparoscopic appendectomy is also associated with decreased rates of wound contamination. The only downside to laparoscopic appendectomy may be that the earliest research on laparoscopic appendectomy suggested a higher rate of infection when this procedure was used for perforated appendicitis [9-11].

Considering the dearth of local literature, the present study was conducted to compare the outcomes of laparoscopic appendectomy versus open appendectomy in our population with perforated appendicitis. 

## Materials and methods

A comparative study was conducted at the Department of Surgery, Liaquat University of Medical & Health Sciences (LUMHS), between March 2, 2019 and July 7, 2020. The LUMHS ethical committee authorized the conduction of this study (Reference # IRB/Surg/5412). Participants were selected with a non-probability convenience sampling technique and data collection commenced.

The inclusion criteria consisted of a diagnosis of perforated appendicitis evidenced by the following symptoms, signs, and investigation findings: severe right iliac fossa pain and tenderness, abdominal rigidity, rebound tenderness, fever, white blood cell (WBC) count > 15,000/mm^3^, and imaging (ultrasound or CT scan) showing periappendicular fluid collection.

Exclusion criteria consisted of intellectual disability hindering the procurement of informed consent, pediatric patients < 15 years of age, patients with an appendicular mass or abscess unsuited for laparoscopic appendectomy, severe sepsis or septic shock on presentation, pregnant women, and non-consenting individuals.

Patients were allocated to either open appendectomy (Group A) or laparoscopic appendectomy (Group B). The patients were allocated to their respective groups using a non-probability consecutive technique. Participants were thoroughly counseled regarding the complications, risks, and advantages of both interventions.

Informed verbal and written consent was procured from the patients in both groups. 

Interventions for all participants were carried out with general anesthesia (GA). Single-dose prophylactic antibiotic cover was administered to all participants during induction of anesthesia. Antibiotic medication used was 1 g of intravenous cefazolin.

All of the surgeries were conducted by an experienced surgical consultant with an experience of at least five years. 

Laparoscopic appendectomy was performed with a standard three-port technique using the Hasson method to develop pneumoperitoneum. Electrocautery was used to dissect the mesoappendix. The appendicular base was knotted and separated between two Ethicon endo-loops with laparoscopic scissors. The dissected tissue was extracted with an extraction bag. The resulting appendicular stump was not regularly buried. Open appendectomy was carried out in a standard fashion making use of the Gridiron incision. The ligation was performed on the mesoappendix. After that, the appendicular base was divided and the tissue was extracted. The appendicular stump was not buried. All the collected tissue samples underwent microscopic investigation.

Postoperatively, regular abdominal auscultation for bowel sounds was conducted twice daily, i.e., 12 hourly. Clear liquid diet was allowed once bowel sounds were audible. Once clear liquid diet was tolerated and passing of flatus had been documented, the diet was progressed to regular. Once regular diet was tolerated and patients remained afebrile for 24 hours, they were discharged.

On discharge participants were required to follow-up for weekly consultations for three weeks. On the first weekly follow-up, stitches were removed. Patients were monitored for complications and adverse events in the following appointments.

The data gathering proforma recorded demographics, surgical findings, operating room (OR) time, length of inpatient care, requirement of analgesic, and any adverse events following surgery.

Statistical Package for the Social Sciences (SPSS) v. 26.0 was utilized to assess the data. Frequencies and percentages were determined for categorical parameters. Categorical variables were compared using the chi-square test. Continuous variables (two-tailed) were compared using the t test. A p value of < 0.05 was established as statistically significant.

## Results

A total of 85 patients were included in the laparoscopic appendectomy group, while 101 cases were included in the open appendectomy group. Demographically, there was no difference between the patient characteristics between the two groups (Table 1).

**Table 1:** Demographic information related to laparoscopic appendectomy versus open appendectomy groups

Parameters	Laparoscopic Appendectomy group (n=85)	Open Appendectomy group (n=101)	p-value
Age Groups (years)			0.99
18-30 years	15 (17.6%)	18 (17.8%)	
30-45 years	31 (36.5%)	37 (36.6%)	
46-60 years	29 (34.1%)	34 (33.7%)	
> 60 years	10 (11.8%)	12 (11.9%)	
Body Mass Index (kg/m^2^)			
Underweight	8 (9.4%)	10 (9.9%)	0.99
Normal	39 (45.9%)	47 (46.5%)	
Overweight	25 (29.41%)	29 (28.71%)	
Obese	13 (15.29%)	15 (14.85%)	
Gender			
Female	44 (51.76%)	52 (51.49%)	0.97
Male	41 (48.24%)	49 (48.51%)	
Mean length of symptoms (days)	5.55 ± 3.2	5.48 ± 2.4	0.578

Table 2 illustrates that the use of analgesics thrice a day to manage the postoperative pain was significantly associated with the open appendectomy (p < 0.0001). Moreover, the postoperative length of hospitalization was significantly higher in the open appendectomy group than the laparoscopic appendectomy group (p < 0.0001). Postoperative pain at four hours and at the time of discharge did not significantly alter between the groups. 

**Table 2:** Postoperative outcomes in  laparoscopic appendectomy versus open appendectomy group

Postoperative Outcomes	Laparoscopic Appendectomy group (n=85)	Open Appendectomy group (n=101)	p-value
Visual analog pain score			
Postoperative fourth hour	9.6 ± 3.22	10.51 ± 5.3	0.623
At discharge	3.44 ± 1.2	4.09 ± 1.01	0.799
Use of painkiller postoperatively (thrice a day)			
Yes	25 (29.4%)	68 (67.3%)	< 0.0001
No	60 (70.6%)	33 (32.7%)	
Hospital stay (days)			< 0.0001
3-5 days	64 (75.3%)	35 (34.7%)	
> 5 days	21 (24.7%)	66 (65.3%)	

About 10.6% patients in the laparoscopic appendectomy group and 5% in the open appendectomy group suffered from bleeding, intraoperatively. The incidence of intraoperative complications did not significantly differ between the groups as seen in Table 3. Wound related complications were significantly lower in patients who underwent laparoscopic appendectomy than those who underwent open appendectomy (23.53% versus 40.5%; p = 0.013).

**Table 3:** Complication rates in laparoscopic appendectomy group versus open appendectomy group

Complications	Laparoscopic Appendectomy group (n=85)	Open Appendectomy A group (n=101)	p-value
Intraoperative complications			
Excessive bleeding	9 (10.6%)	5 (5%)	0.146
Ileal injury	1 (1.18%)	0 (0%)	0.457
24 hours postoperative	9 (10.6%)	5 (5%)	0.147
30 days postoperative			
Chest infection	18 (21.18%)	18 (17.82%)	0.537
Ileus	13 (15.29%)	27 (26.73%)	0.058
Intra-abdominal abscess (IAA)	10 (11.76%)	5 (4.95%)	0.089
Wound-related complications	20 (23.53%)	41 (40.5%)	0.013

## Discussion

Mariage M et al. describe perforated appendicitis as a feature of complicated appendicitis [12]. A recent analysis of three randomized-control trials by Quah GS et al. showed that whilst open appendectomy is currently the more common procedure performed for complicated appendicitis due to a reported higher incidence of intra-abdominal abscess (IAA) formation with laparoscopic appendectomy, the latter demonstrates a statistically significant decrease in death and disability [13]. Laparoscopic appendectomy is also associated with a shorter length of inpatient stay and better health outcomes when contrasted with open appendectomy. They also found similar statistics of IAA between both groups. The researchers subsequently recommended laparoscopic appendectomy for complicated appendicitis. This is in contradiction to our findings that showed no statistically significant benefit of laparoscopic appendectomy over open appendectomy in terms of postoperative health outcomes. However, after the three-to-five-day hospital stay, incidence of IAA and mortality, though not statistically significant, were higher in the laparoscopic appendectomy group [13].

Horvath P et al. conducted a retrospective study on 1,762 patients comparing laparoscopic appendectomy to open appendectomy for perforated appendicitis [14]. They found that while postoperative complications like SSIs only occurred in patients who underwent open appendectomy, the occurrence of IAA in patients who underwent laparoscopic appendectomy was statistically significant with a p-value of 0.002. They also reported shorter inpatient stay after laparoscopic appendectomy. In this study, it was advised that surgeons keep in mind the steps that can be taken to reduce the formation of IAA such as irrigations, handling of the stump, and use of endo bags [14].

In a meta-analysis conducted by Athanasiou C et al., it was demonstrated that, as repetitively evidenced in the literature, SSIs, length of inpatient stay, and early tolerance of oral diet were all significantly lower after laparoscopic appendectomy [15]. Like Quah GS et al. [13], they found no statistically significant difference between incidences of IAA after both procedures. Thus, it was reported that laparoscopic appendectomy has better outcomes for morbidity [15].

Yu MC et al. also produced results in their meta-analysis and systematic review favoring laparoscopic appendectomy over open appendectomy in complicated appendicitis. IAA was also seen to not increase with laparoscopic appendectomy in this study [16].

This recurrent statistic of higher incidence of IAA after laparoscopic appendectomy may be better understood by an analysis of the risk factors for IAA. Schlottmann F et al. described higher incidence of IAA after laparoscopic appendectomy in the following patient groups: obese patients, patient with a WBC count > 20,000/mm^3^, and maintenance of pneumoperitoneum for longer times. Obesity is related to metabolic and thus immune dysfunction, and a higher WBC count indicates more severe infection and pathology. This may explain their association with laparoscopic appendectomy. They also identified the perforated appendix, i.e., greater extent of pathology itself as a risk factor for IAA [17]. Thus, aside from non-modifiable patient factors, surgeon technique may be beneficial in reducing the incidence of IAA post-laparoscopic appendectomy. 

The above results were replicated in a retrospective study on the risk factors for IAA after laparoscopic appendectomy in acute uncomplicated appendicitis (UA). Fernández-Moreno MC et al. demonstrated that laparoscopic appendectomy is not associated with greater risk of IAA. The risk factors for IAA involve factors relating to poorer immune functioning (i.e., diabetes mellitus) and more profound infection (i.e., high c-reactive protein (CRP)) [18]. Thus, IAA may be independent of laparoscopic appendectomy.

Wullstein C et al. retrospectively analyzed laparoscopic appendectomy vs open appendectomy for perforated appendicitis and found laparoscopic appendectomy better in terms of patient outcomes [19]. Ball CG et al. recommended laparoscopic appendectomy as the procedure of choice for complicated appendicitis [20].

Mulita F et al. retrospectively observed the outcomes of laparoscopic appendectomy and open appendectomy on patients suffering from both complicated appendicitis and UA and found that regardless of the type of appendicitis or the technique of appendectomy the incidence of IAA does not significantly vary. They advised the preference of laparoscopic appendectomy over open appendectomy because of the benefits that minimally invasive laparoscopy provides [21]. In some rare cases, diagnosis become quite challenging thus delaying the treatment. For instance, a case report revealed an 18-year-old female who had acute lymphoblastic leukemia and presented with acute appendicitis. The patient immediately underwent open appendectomy and had no intra or postoperative complications [22]. However, in the present study, none of the patients had leukemia or any other malignancy. 

In this study, the statistically significant benefits of laparoscopic appendectomy over open appendectomy were reduced time to introduction of oral diet, shorter course of antibiotics, lower need for analgesia, and early drain removal. Other benefits noticed were reduced SSIs and lower incidence of paralytic ileus. Our study indicated that three-to-five days of inpatient care were higher in the laparoscopic appendectomy group but more than five days of inpatient care was more common in the open appendectomy group, suggesting that occasionally, open appendectomy did result in earlier discharge unless postoperative complications occurred, the likelihood of which was high.

Our study, in light of the literature, suggests that laparoscopic appendectomy may very well be a more improved and safe procedure in terms of health outcomes granted that proper technique, stratification of patient risk factors, and postoperative care are guaranteed. Nonetheless, the scope of this study does not facilitate a more in-depth analysis and further research is warranted in order to impact policies.

## Conclusions

The present study revealed that the length of stay was significantly lower in patients who underwent laparoscopic appendectomy. Furthermore, we also found that laparoscopic appendectomy was significantly correlated with less frequency of wound infections postoperatively. Therefore, in the light of current evidence and the literature review we can conclude that laparoscopic appendectomy yields more favorable outcomes than the open appendectomy. 
